# Microscopy image reconstruction with physics-informed denoising diffusion probabilistic model

**DOI:** 10.1038/s44172-024-00331-z

**Published:** 2024-12-29

**Authors:** Rui Li, Gabriel della Maggiora, Vardan Andriasyan, Anthony Petkidis, Artsemi Yushkevich, Nikita Deshpande, Mikhail Kudryashev, Artur Yakimovich

**Affiliations:** 1 Center for Advanced Systems Understanding (CASUS), Görlitz, Germany; 2 Helmholtz-Zentrum Dresden-Rossendorf e. V. (HZDR), Dresden, Germany; 3 Department of Molecular Life Sciences, University of Zurich, Zurich, Switzerland; 4 In situ Structural Biology, Max Delbrück Center for Molecular Medicine in the Helmholtz Association, Berlin, Germany; 5 Institute of Medical Physics and Biophysics, Charite-Universitätsmedizin, Berlin, Germany; 6 Institute of Computer Science, University of Wrocław, Wrocław, Poland

**Keywords:** Image processing, Optical physics

## Abstract

Light microscopy is a practical tool for advancing biomedical research and diagnostics, offering invaluable insights into the cellular and subcellular structures of living organisms. However, diffraction and optical imperfections actively hinder the attainment of high-quality images. In recent years, there has been a growing interest in applying deep learning techniques to overcome these challenges in light microscopy imaging. Nonetheless, the resulting reconstructions often suffer from undesirable artefacts and hallucinations. Here, we introduce a deep learning-based approach that incorporates the fundamental physics of light propagation in microscopy into the loss function. This model employs a conditioned diffusion model in a physics-informed architecture. To mitigate the issue of limited available data, we utilise synthetic datasets for training purposes. Our results demonstrate consistent enhancements in image quality and substantial reductions in artefacts when compared to state-of-the-art methods. The presented technique is intuitively accessible and allows obtaining higher quality microscopy images for biomedical studies.

## Abstract (web summary)

Rui Li, Gabriel della Maggiora and co-authors present a deep learning approach for attenuating diffraction and optical imperfections in light microscopy images. By incorporating the underlying physics of light propagation in microscopy into the loss function and designing a conditional diffusion model, they obtained improved performance compared to the state-of-the-art.

## Introduction

**Fig. 1:**
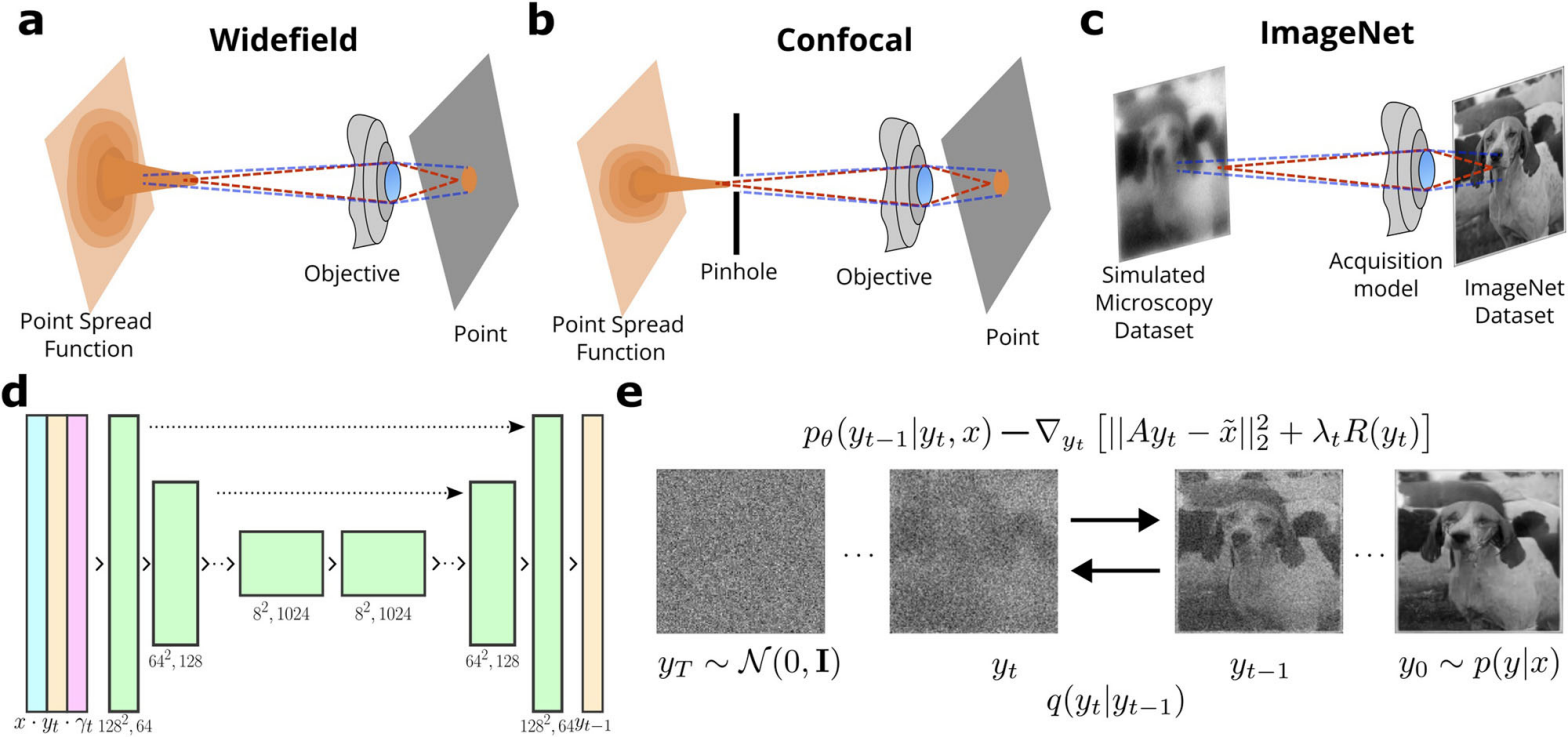
Proposed model with physics-informed probabilistic denoising diffusion for Microscopy image reconstruction. **a**, **b** simplified schematic depiction of widefield and confocal microscopy. **c** Schematic depiction of synthetic dataset generation using our image acquisition model. **d** Schematic depiction of our model’s architecture. Downsampling green blocks are convolution blocks with instance normalisation. Upsampling green blocks have an additional bilinear upsampling operator. Yellow blocks represent the diffusion state. Blue blocks represent the widefield image and red blocks represent the *γ* function at timestep *t* (**e**) illustration of the physics-informed denoising diffusion probabilistic model we propose. The diffusion process is carried out by distribution *q*, which progressively destroys the data structure until reaching a *N*(0, **I**) distribution. The reverse process progressively recovers the structure of the data. This process is characterised by the learned distribution *p*, in which we incorporate the physics model to inform the recovery of the data (See Methods). The physics model is assumed to be linear, represented by matrix *A*, and can also incorporate a regulariser *R*, such as *L*_1_ or *L*_2_.

Light microscopy (LM) is an important and accessible way to explore the hidden biomedical world of cells and sub-cellular structures. The decreasing cost of basic LM equipment is facilitating their accessibility in classrooms for educational purposes^1–4^ and in medical laboratories, particularly for applications such as cytometry^1^. Moreover, it is hard to overstate the significant contributions made by advanced LM techniques such as fluorescence microscopy^5^, confocal microscopy^6^, or superresolution microscopy^7^ to a myriad of biomedical discoveries over the past century^8^. Yet, optical systems remain fundamentally limited owing to the principles of their design (Fig. 1a, b). The blur and imperfections of a point source (e.g. single molecule fluorescence) can be expressed mathematically as a point spread function (PSF)^9^. PSF describes the spread of light that occurs from scattering and diffraction as it passes through the optical components of the microscope. The advent of digital microscopy and image processing allowed us to attempt alleviating these limitations algorithmically^10–15^. These methods include deconvolution methods^9,16,17^, regularisation methods^18–20^ and bayesian methods^21^. However, the traditional methods fail to capture the complexity of the images. This leads to minuscule resolution improvement in reconstructions. Moreover, susceptibility to noise and artefacts during image acquisition can compromise the performance of these methods, resulting in degraded quality during reconstruction.

The deep learning (DL) models, particularly convolutional neural networks (CNNs), have gained traction in image reconstruction tasks^22–25^. CNNs learn complex mappings between images and their corresponding ground truth. For example, Xu et al. transformed a pseudo-inverse kernel for deconvolution into a CNN, capturing degradation characteristics^26^. Ronneberger et al. incorporated deconvolution layers into the U-Net architecture^27^. Generative adversarial networks have also been used for image restoration^28,29^.

Another recent trend involves the utilisation of a type of likelihood-based generative models known as Denoising Probabilistic Diffusion Models (DDPMs)^30^. They have shown promise in several tasks such as superresolution^31^, image colourisation, inpainting, uncropping, and JPEG restoration^32^. The diffusion models have desirable properties such as distribution coverage, stationary optimisation function, scalability, and training stability. They demonstrate promising results in terms of generating high-quality samples when compared to generative adversarial networks (GANs)^33^.

However, most of these approaches ignore the great body of knowledge gathered on microscopy by optical physics. One common challenge in DL methods involves their inclination to produce structures not found in real images. This poses issues in fields that demand accurate reconstruction, such as medical diagnostic images and microscopy^34,35^. To circumvent this, in other domains where the physics of the process is well understood, researchers have recently proposed an approach called physics-informed neural networks^36^. In this approach, the prior knowledge regarding the physics models serves as a regularisation for DL models. While other approaches exist, incorporating physics by using the physical model as a regulariser is intuitive and widely used.

To overcome the shortcomings, we introduce a physics-informed (PI) diffusion model (Fig. 1d, e). In this model, the PI term is incorporated in the DDPM loss function (see Fig. 1e, Methods) using the technique shown in refs. ^33,37^. We show that this approach not only provides a simpler and more principled model but also produces more natural-looking results.

## Results

### Light microscopy image acquisition model

LM is an imaging technique in which an analogue-digital converter detects the electrical impulses generated by the light. As a result, image statistics can be well represented by a Poisson process. If several acquisitions are made and averaged, then according to the central limit theorem, the statistics of the image can be modelled by the Gaussian process. The mathematical model for optical systems assumes that the model is linear and time-invariant. Therefore the image acquisition model is described by the equation *I* = *ϕ*(*h*∗*x* + *b*), where the image *I*, is the result of the convolution between object *x* and system PSF *h* with the background signal noise *b* added. The Poisson noise *ϕ* is applied afterwards over the true signal, given by the previous equation.

In LM, the diffraction pattern generated in an ideal optical system is the impulse response referred to as the PSF^9^. PSF of LM varies depending on the specifics of the technique employed, e.g. widefield and confocal LM (Fig. 1a, b). The diffraction pattern generated in an ideal optical system is the impulse response referred to as the PSF. In fluorescence microscopy, usually, the illumination (excitation) and detection (emission) wavelength are not the same, so the most suitable model of the PSF^38^ can be expressed as:

1 $$h(x,y,z)=| {u}_{{\lambda }_{{{{\rm{ex}}}}}}(x,y,z){| }^{2}| {u}_{{\lambda }_{{{{\rm{em}}}}}}(x,y,z){| }^{2},$$

where *u*_*λ*_ corresponds to the pupil function for a respective emission or excitation wavelength *λ*. This model is known as the Airy diffraction pattern^39^. To add the effect of the pinhole used in confocal microscopy, we can convolve a disk function with the pupil of the emission sample. The disk function is usually modelled as:

2 $$T(x)={1}_{{R}^{2}\le {X}^{2}+{Y}^{2}},$$

where R is the radius of the pinhole. Using this notion, we can rewrite the PSF as:

3 $$h(x,y,z)=| T(x,y)* {u}_{{\lambda }_{{{{\rm{em}}}}}}(x,y,z){| }^{2}| {u}_{{\lambda }_{{{{\rm{ex}}}}}}(x,y,z){| }^{2}.$$

To model the pupil function *u* above, we employed the Arnison-Sheppard approach^40^. This approach models the optical transfer function (OTF). The OTF is expressed as the autocorrelation of the pupil function *u* in the Fourier space. Thus, the *u* is the inverse Fourier transform of *C*(**K**). Mathematically, in the k-space the OTF *C*(**K**) can be denoted as below:

4 $$C({{{\bf{K}}}})={\iint} {\int} Q({{{\bf{m}}}}+\frac{1}{2}{{{\bf{K}}}})\cdot {Q}^{* }({{{\bf{m}}}}-\frac{1}{2}{{{\bf{K}}}})d{{{\bf{m}}}},$$

where Q corresponds to the complex vectorial pupil function^41^.

The complex vectorial pupil function is a complex-valued function *Q*(**m**) of the position vector **m** = (*k*_*x*_, *k*_*y*_, *k*_*z*_) within the aperture of an optical system. This function can be expressed as:

5 $$Q({{{\bf{m}}}})=A({{{\bf{m}}}}){e}^{i\phi ({{{\bf{m}}}})},$$

where *A* is the amplitude transmission function with respect to the numerical aperture of the microscope and *ϕ* is the phase shift produced by aberrations and microscope imperfections. Finally, to obtain *u* the inverse Fourier transform is applied to the OTF.

### Physics-informed denoising probabilistic diffusion models

Physics-informed methods arise from the need to incorporate physical knowledge into the construction or training of the model. In inverse problems, the objective is to determine *p*(*y*∣*x*) where *y* is the true object and *x* is some observation of the object that follows a possibly stochastic process *p*(*x*∣*y*). In image reconstruction problems such as MRI undersampled image reconstruction, deblurring or microscopy image reconstruction, the physical model is generally of the form *x* = *ϕ*(*K*∗*y*) where *ϕ* is a function that applies noise according to a certain distribution. Particularly, model-based methods try to solve a problem of the form ${\min }_{y}| | Ay-x| {| }^{2}+\lambda R(y).$ Where *λ* is the weight of the regulariser *R*(*y*) and *A* is a matrix that represents the convolution operation with the PSF *K*.

To recover the true object from the observed variables. This formulation can be incorporated into the model-guided DDPM^42^ (See Methods for details on Denoising Diffusion Probabilistic Models and Conditioned Denoising Probabilistic Diffusion models, as well as Supplementary Materials) to obtain improvements over the conditioned case by using a physical prior. However, one cannot access *K* during inference in several cases. To remediate this, we suggest learning the shifted mean of a guided DDPM by the physical model. In this sense, we incorporate the gradient of the solution of the physical problem as a shift of the mean of the unconditioned case.

We describe the PI Model based on the framework of Guided Diffusion Models^33^. We recall that the gradient of the data likelihood denoted as $g={\nabla }_{{y}_{t}}\log p(x| {y}_{t})$ guides the sampling through the chain. We approximate the transition *p*(*y*_*t*_∣*y*_*t*+1_)*p*(*x*∣*y*_*t*_) with the expression:

6 $$p({y}_{t}| {y}_{t+1})p(x| {y}_{t})\approx {{{\mathcal{N}}}}({y}_{t};\mu +{\sigma }_{t}^{2}g,{\sigma }_{t}^{2}I)$$

For our specific problem of Microscopy image reconstruction, the conditioning variable *x* represents the measured data and follows the measurement model *x* = *Ay* + *n*, *n* ~ *N*(*n*; *ξ*, *ξI*), and *A* represents the convolution operation with the microscope PSF. Therefore, considering the posterior of the forward process *q*(*y*_*t*_∣*y*_*t*+1_, *y*_0_), we express the target posterior as:

7 $$q({y}_{t}| {y}_{t+1},{y}_{0},x)\approx q({y}_{t}| {y}_{t+1},{y}_{0})p(x| {y}_{t})\approx {{{\mathcal{N}}}}({y}_{t};\widetilde{\mu }+{\sigma }_{t}^{2}g,{\sigma }_{t}^{2}I)$$

Here, $\widetilde{\mu }$ is defined as $\widetilde{\mu }=\frac{\sqrt{{\gamma }_{t-1}}{\beta }_{t}}{1-{\gamma }_{t}}{y}_{0}+\frac{\sqrt{{\alpha }_{t}}(1-{\gamma }_{t-1})}{1-{\gamma }_{t}}{y}_{t}$.

To train the transition *p*_*θ*_(*y*_*t*_∣*y*_*t*+1_, *x*), analogous to regular DDPMs, we define ${p}_{\theta }({y}_{t}| {y}_{t+1},x)={{{\mathcal{N}}}}({y}_{t};{\mu }_{\theta }({y}_{t},x),{\sigma }_{t}^{2}I)$, and optimise it using the following mean squared error loss:

8 $${L}_{{{{\rm{mean}}}}}:= {\mathbb{E}}\left[\frac{1}{2{\sigma }_{t}^{2}}| | \widetilde{\mu }({y}_{0},{y}_{t})+{\sigma }_{t}^{2}g-{\mu }_{\theta }({y}_{t},x)| {| }_{2}^{2}\right]$$

Using the reparametrisation trick, we can rewrite the loss function as:

9 $${\mathbb{E}}\left[\frac{1}{2{\sigma }_{t}^{2}}{\left| \left| \frac{1}{\sqrt{{\alpha }_{t}}}\left({y}_{t}({y}_{0},x,\epsilon )-\frac{{\beta }_{t}}{\sqrt{1-{\gamma }_{t}}}\epsilon \right)+{\sigma }_{t}^{2}g+{\mu }_{\theta }({y}_{t},x)\right| \right| }_{2}^{2}\right]$$

Here, we reparametrise the neural network prediction with *ϵ*_*θ*_(*x*, *y*_*t*_), leading to:

10 $${\mathbb{E}}\left[\frac{1}{2{\sigma }_{t}^{2}}{\left| \left| \frac{1}{\sqrt{{\alpha }_{t}}}\left({y}_{t}({y}_{0},x,\epsilon )-\frac{{\beta }_{t}}{\sqrt{1-{\gamma }_{t}}}\epsilon \right)+{\sigma }_{t}^{2}g\\ -\frac{1}{\sqrt{{\alpha }_{t}}}\left({y}_{t}({y}_{0},x,\epsilon )-\frac{{\beta }_{t}}{\sqrt{1-{\gamma }_{t}}}{\epsilon }_{\theta }(x,{y}_{t})\right)\right| \right| }_{2}^{2}\right]$$

To further simplify, we introduce a factorisation term $C=\frac{{\beta }_{t}^{2}}{2{\sigma }_{t}^{2}{\alpha }_{t}(1-{\gamma }_{t})}$, resulting in:

11 $${L}_{{{{\rm{pi}}}}{\mbox{-}}{{{\rm{simple}}}}}={{\mathbb{E}}}_{\epsilon ,t,(x,{y}_{0})}\left[C{\left| \left| \epsilon -{\epsilon }_{\theta }(\sqrt{{\gamma }_{t}}{y}_{0}+\sqrt{1-{\gamma }_{t}}\epsilon ,x,{\gamma }_{t})+\frac{{\sigma }_{t}^{2}}{\sqrt{C}}g\right| \right| }_{2}^{2}\right]$$

We use a simplification of the loss function and set *C* = 1 to train our model.^30^ One remaining challenge is defining the gradient term ${\nabla }_{{y}_{t}}\log p(x| {y}_{t})$. In the context of linear inverse problems, particularly in linear inverse problems tasks, such as microscopy image super-resolution, that solve the following optimisation problem:

12 $${\min }_{y}| | Ay-x| {| }_{2}^{2}+\lambda R(y),$$

several approaches have been proposed to approximate ${\nabla }_{{y}_{t}}\log p(x| {y}_{t})$. One line of work suggests an approximation of ${\nabla }_{{y}_{t}}\log p(x| {y}_{t})\approx \frac{{A}^{T}(x-A{y}_{t})}{{\sigma }^{2}}$ ^43^. This approximation assumes white Gaussian noise in the measurement process, with variance *σ*^2^, and utilises the conversion of the convolution operation into its matrix representation *A*.

Another approach aims to approximate the gradient by evaluating the measurement model in the model estimation at timestep *t*^42^. Specifically, they define that the distribution *p*(*x*∣*y*_*t*_) can be approximated in the following manner:

13 $$p(x| {y}_{t})\approx p(x| \widehat{{y}_{0}})$$

Where, $\widehat{{y}_{0}}={\mathbb{E}}[{y}_{0}| {y}_{t}]$, and can be sampled with $\widehat{{y}_{0}}=\frac{{y}_{t}-\sqrt{1-{\gamma }_{t}}{\epsilon }_{\theta }({y}_{t},x,{\gamma }_{t})}{\sqrt{{\gamma }_{t}}}$.

While this second approximation exhibits improved accuracy over the first one, it introduces a dependency on the model prediction, thereby generating a learning gradient through the function *g*. As our method seeks to avoid any learning signal through the function *g*, we find the second approximation unsuitable for our purposes. Introducing a dependency on model predictions may lead to deviations from the intended objective of learning the shifted mean of the forward process posterior.

We adopt the ${\nabla }_{{y}_{t}}\log p(x| {y}_{t})\approx \frac{{A}^{T}(x-A{y}_{t})}{{\sigma }^{2}}$ approximation and adapt it to account for noise following a distribution *n* ~ *N*(*n*; *ξ*, *ξI*), where the measurement is given by *x* = *Ay* + *n*. Under this measurement model the approximation of ${\nabla }_{{y}_{t}}\log p(x| {y}_{t})$ is

14 $${\nabla }_{{y}_{t}}\log p(x| {y}_{t})\approx \frac{{A}^{T}(x-\xi -A{y}_{t})}{{\xi }^{2}}$$

Since we train with synthetic data, we can access the noiseless version of the measurement, enabling us to replace *x* − *ξ* with its noiseless counterpart. Using the same strategy described in^43^, we employed annealing on the gradient of Eq. (14) with a variable *ν*_*t*_ that increases as *t* → 0. Then the final gradient term is:

15 $${\nabla }_{{y}_{t}}\log p(\widetilde{x}| {y}_{t})\approx {\nu }_{t}{\nabla }_{{y}_{t}}| | A{y}_{t}-\widetilde{x}| {| }_{2}^{2}$$

We find in our experiments that the best results were obtained with ${\nu }_{t}=\eta \frac{\sqrt{{\gamma }_{t}}}{| | {\nabla }_{{y}_{t}}\log p(\widetilde{x}| {y}_{t})| {| }_{2}}$, where *η* is set to 10. Detailed algorithms of training and sampling are provided in the Methods section in Algorithm 1 and Algorithm 2 respectively.

### Training dataset

**Fig. 2:**
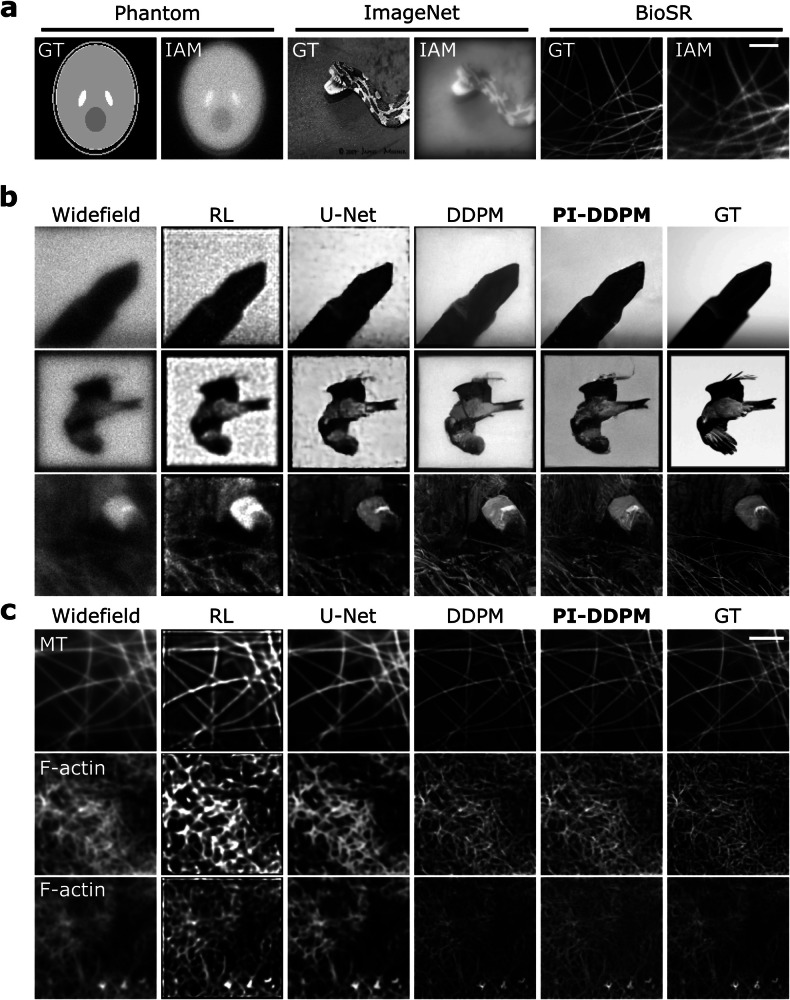
Proposed model with physics-informed probabilistic denoising diffusion for Microscopy image reconstruction. **a** Examples of processing performed using Image Acquisition Model (IAM) on (left-to-right) Shepp-Logan phantom, ImageNet and BioSR images. Simulated examples. **b** Examples of input (Widefield) and reconstructed images using Richardson-Lucy (RL), U-Net, Denoising Diffusion Probabilistic Models (DDPM) and physics-informed DDPM (PI-DDPM) in ImageNet and BioSR-derived images, respectively. Simulated dataset based on ImageNet dataset. **c** Denoising Diffusion Probabilistic Models (DDPM) and Physics-informed DDPM (PI-DDPM) in BioSR images. BioSR is a dataset of real widefield and SIM micrographs. GT stands for ground truth. MT stands for microtubules. Scale bar in micrographs is 2 *μ*m.

To ensure that a large dataset is available for training, we simulated the microscopy images (see Fig. 1c, Methods). For this, we have processed images derived from two large datasets ImageNet^44^ and the biological image dataset for super-resolution microscopy (BioSR)^29^ using our image acquisition model (IAM, see Methods, Supplement). Changes that such processing inflicts on the images are demonstrated in Figs. 1c and  2a. These images used for training bore a strong resemblance to images obtained using LM.

### Model training and benchmark evaluation

Once our PI Denoising Diffusion Probabilistic Model (PI-DDPM, see Fig. 1d, e, Methods) was trained, we compared the PI-DDPM model’s performance against other mainstream models, including U-Net, DDPM, and the Richardson-Lucy (RL) algorithm (Fig. 2b, c, Sup. Table 1). For this purpose, we trained U-Net and DDPM alongside PI-DDPM and included RL for comparison. Our visual assessment indicated that both DDPM and PI-DDPM exhibited fewer noise and processing artefacts than RL and U-Net. Notably, PI-DDPM excelled in preserving high-frequency details in both ImageNet and BioSR-derived images. To quantify this difference, we evaluated the performance with three metrics: peak single-to-noise ratio^45^, multi-scale structural similarity index measure (MS-SSIM)^46^, and normalised root mean square error (NRMSE). The results demonstrate the superiority of DDPM and PI-DDPM in image restoration (Table 1, BioSR, Sup. Table 1). Furthermore, applying the models to microscopy images from the BioSR test set further established the superiority of both DDPM and PI-DDPM over U-Net across all metrics (Table 1, BioSR). Notably, PI-DDPM improved over DDPM in this more realistic scenario. Additionally, we visually compared the reconstruction results on the BioSR dataset between DDPM and its PI counterpart proposed here. Crucially, DDPM hallucinated non-existent structures, while PI-DDPM remained close to the GT (Sup. Fig. S1a)

Next, to evaluate the model on another benchmark dataset we have employed widefield to structured illumination microscopy (W2S) benchmark^47^. Results suggested that the performance of our PI-DDPM was superior over DDPM and U-Net in PSNR, MS-SSIM and NRMSE metrics (Table 1, W2S).

To expand our choice of metrics, we have computed Fourier ring correlation (FRC) and decorrelation-based resolution^48^ on the individual subcellular structures of the BioSR dataset. These structures included clathrin-coated pits (CCP), endoplasmic reticulum (ER), microtubules (MT) and F-actin. Furthermore, we expanded our comparison to the GAN-based state-of-the-art specialist SR models including DFGAN^28^ and DFCAN^29^ (Sup. Fig. S1b). Results suggested that while the specialist DFGAN model often performed best, PI-DDPM performed second-best outperforming the baseline DDPM. Remarkably, when the FRC of high-resolution details was compared on MT and ER, PI-DDPM either outperformed the rest or performed comparably to DFGAN, outperforming the baseline DDPM by a margin.

Finally, we compared how DDPM and PI-DDPM perform in the presence of noise and with only limited data (Sup. Fig. S2). In this experiment, using the F-actin images from the BioSR dataset, we randomly sampled 100 images from the test set and simulated low-quality widefield acquisitions by introducing the increasing amount of controlled Poisson-Gaussian noise. Results suggested that PI-DDPM reconstructions preserved the triangular shape of the filamentous structures of F-actin more reliably than DDPM in high noise conditions (Sup. Fig. S2a, zoomed-in insets). Furthermore, PI-DDPM metrics deteriorated less with increased noise (Sup. Fig. S2b), suggesting higher robustness.

### Model evaluation on non-benchmark superresolution microscopy

**Fig. 3:**
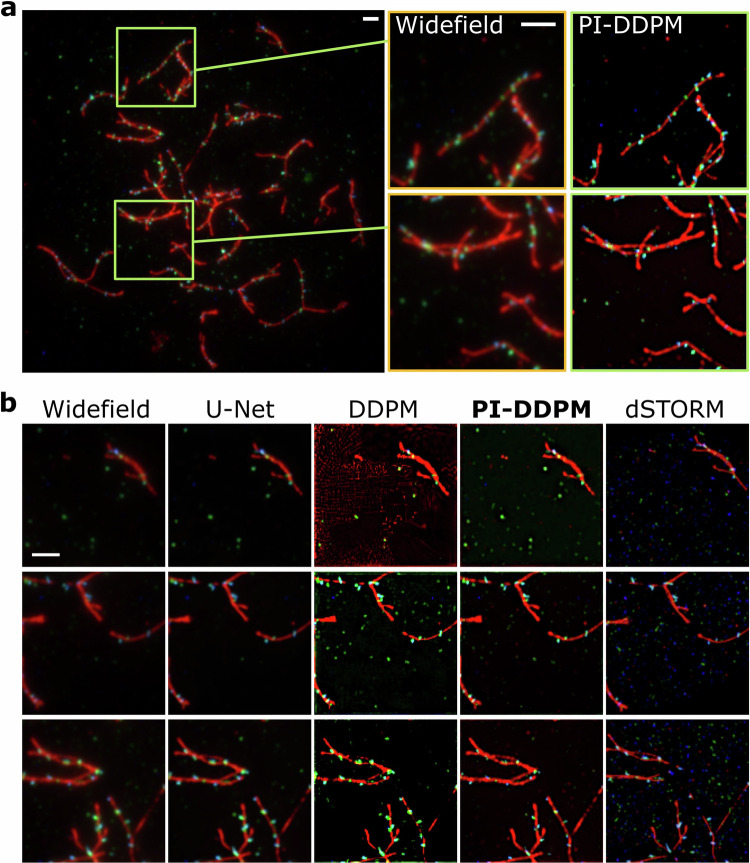
Model performance on unseen superresolution dataset. **a** Image containing mid-zygotene nucleus immunostained for SYCP3 (red), DMC1 (green) and RAD51 (blue) proteins from ref. ^49^. **b** Examples of input (Widefield) and reconstructed images using U-Net, Denoising Diffusion Probabilistic Models (DDPM) and physics-informed DDPM (PI-DDPM) in dSTORM images. The scale bar is 2 *μ*m.

To assess the models’ adaptability, we employed a published non-benchmark real-world microscopy dataset of Direct Stochastic Optical Reconstruction Microscopy (dSTORM)^49^. This dataset was previously unseen by our model and unprocessed. In this dSTORM dataset, authors provide widefield (low-resolution) and dSTORM (superresolved) microscopy paired images containing mid-zygotene nucleus immunostained for SYCP3 (red), DMC1 (green) and RAD51 (blue) proteins. PI-DDPM demonstrated exceptional performance on the dSTORM dataset, producing results closely aligned with the ground truth (Fig. 3a, b, Methods). As previously the quantitative evaluation was performed by computing metrics on images reconstructed from the widefield images and compared the actual dSTORM reconstruction as GT. The model surpassed both U-Net and DDPM by yielding sharper reconstructions and exhibiting fewer artefacts in low-signal scenarios. Remarkably, PI-DDPM produces visibly fewer artefacts in low signal (Fig. 3b, red channel, upper row) compared to DDPM. Additionally, PI-DDPM preserved the continuity of the filament structures better (Fig. 3b, red channel, lowest row) compared to DDPM. Interestingly, DDPM seems to overemphasise the green channel (DMC1) possibly due to the high signal-to-noise. However, all models struggled to capture low signal-to-noise punctae in RAD51 (blue channel). A thorough quantitative evaluation across different channels indicated that both DDPM and PI-DDPM consistently outperformed other models, with PI-DDPM having a slight edge in PSNR and NRMSE metrics and showing comparable results in MS-SSIM to DDPM (Table 1, dSTORM).

### Model evaluation on real-world prospectively acquired microscopy

**Fig. 4:**
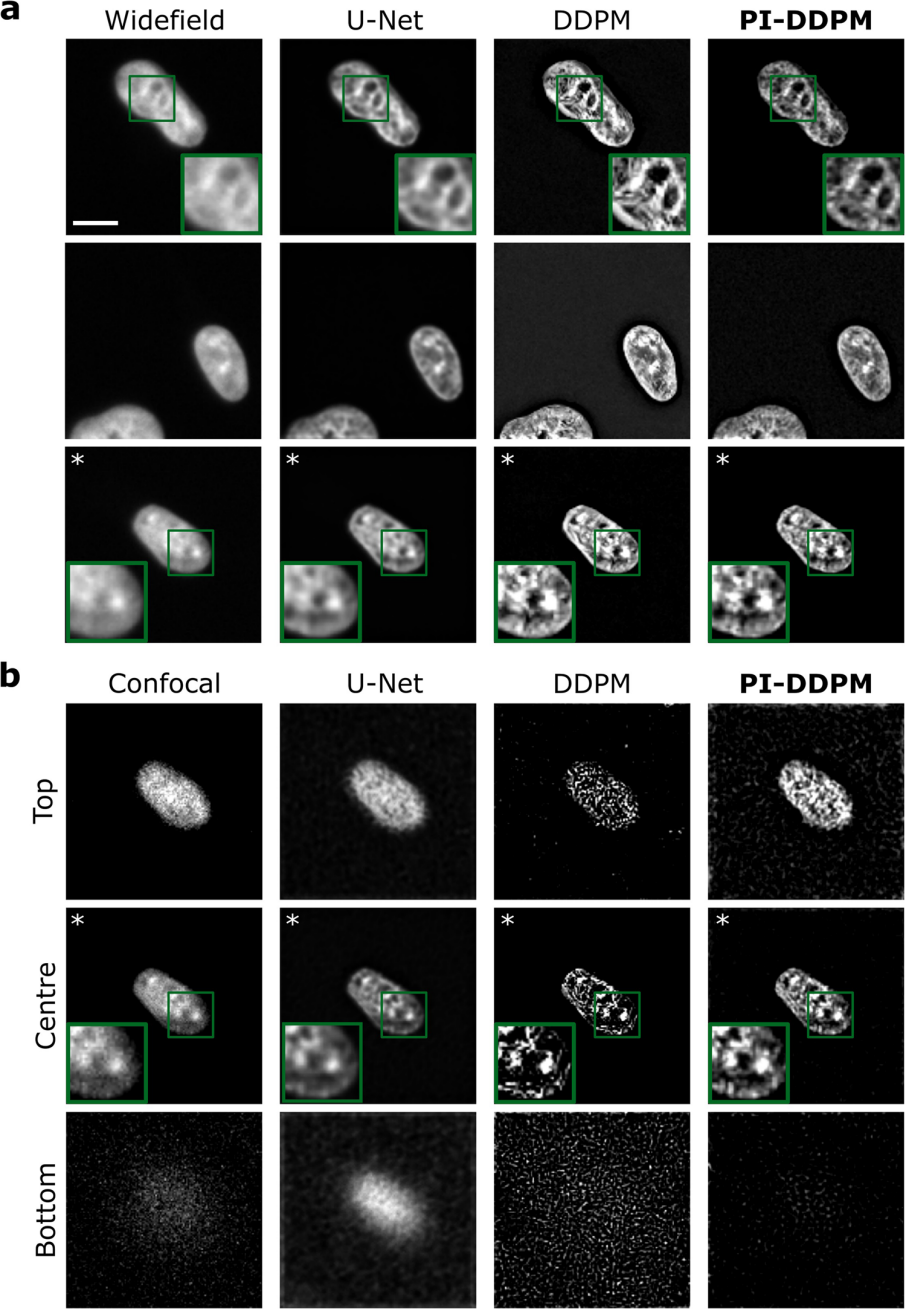
Model performance on prospective correlative widefield-confocal microscopy. **a** Examples of widefield images of cell nuclei and their reconstructions using U-Net, Denoising Diffusion Probabilistic Models (DDPM) and physics-informed DDPM (PI-DDPM). **b** Examples of confocal images and their reconstructions. Top, centre and bottom refer to the Z-positions of the slices. The bottom lies beyond the edge of the specimen. The scale bar is 10 *μ*m. Asterisk (*) marks correlated images of the same cell and focal plane.

We further tested our model using a correlative widefield-confocal microscopy dataset of cell nuclei, which we acquired prospectively to test our model (Fig. 4)^50^. In this dataset, both confocal and widefield stacks of the same region were taken by automated microscopy (see Methods). Since confocal microscopy (Fig. 4b) is known to have better resolution compared to widefield microscopy (Fig. 4a) it can serve as a guide on the correctness of image restoration in the absence of *bona fide* ground truth. Consistent with our previous observations, we noted that DDPM and PI-DDPM have shown significantly lower blur in the reconstructions (Fig. 4a, top row). Remarkably, comparing correlated fields of view (Fig. 4a,b, white asterisk) PI-DDPM reconstructions show more consistency with the confocal image than the conventional DDPM or U-Net irrespective of whether the input image comes from widefield or confocal microscopy. Furthermore, PI-DDPM showed more consistent output in the case of a low signal-to-noise ratio beyond the edge of the specimen(Fig. 4b, bottom).

### Ablation study: the importance of regularisation and parameter *η*

**Fig. 5:**
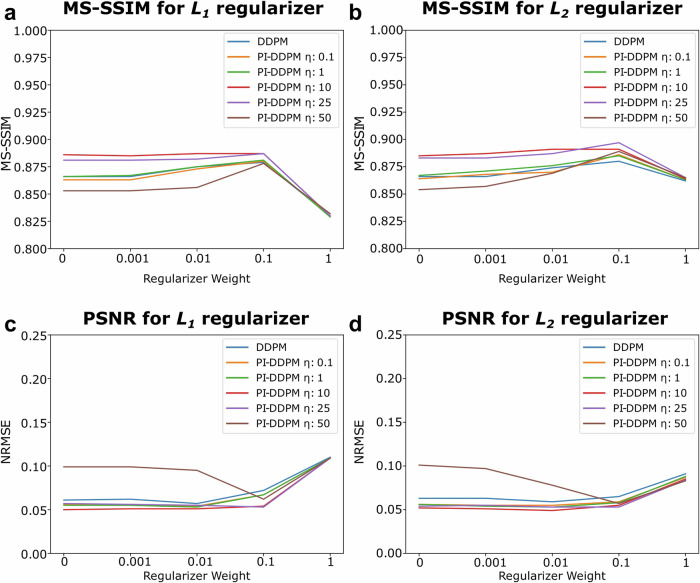
Influence of the parameter *η*, and different regularisers on the learning process. **a** Influence of decreasing *η* measured by Multi-Scale Structural Similarity (MS-SSIM) for *L*_1_ regularisation. **b** Influence Influence of decreasing *η* measured by MS-SSIM for *L*_2_ regularisation. **c** Influence of decreasing *η* measured by the peak signal-to-noise ratio (PSNR) for *L*_1_ regularisation. **d** Influence of decreasing *η* measured by PSNR for *L*_2_ regularisation.

To evaluate the importance of the regularisation term and the parameter *η* in our model, we conducted a series of experiments on a randomly selected subset of the BioSR test dataset (Fig. 5). This ablation study aimed to provide insights into the contributions and effects of these components on the overall performance of our proposed approach. Results are presented in Sup. Table S2 and S3, as well as Fig. 5.

One fundamental aspect under investigation is the impact of the regularisation term applied to the learning process. In the context of conditioned DDPMs (DDPM baseline), we follow the next regularisation formulation:

16 $${\nabla }_{{y}_{t}}\log p({y}_{t})\approx {\nabla }_{{y}_{t}}{\lambda }_{t}R({y}_{t}),$$

where ${\nabla }_{{y}_{t}}\log p({y}_{t})$ denotes the gradient of the log-likelihood with respect to the perturbed latent variable *y*_*t*_, *λ*_*t*_ is the regularisation coefficient at time step *t*, and *R*(*y*_*t*_) represents the regularisation term.

In contrast, our proposed model introduces the learned gradient and the regularisation term as follows:

17 $${\nabla }_{{y}_{t}}\log p(\tilde{x}| {y}_{t})\approx {\nabla }_{{y}_{t}}\left[| | A{y}_{t}-\tilde{x}| {| }_{2}^{2}+{\lambda }_{t}R({y}_{t})\right],$$

where *A* is the linear operator of the forward problem, $\tilde{x}$ represents the measured image without noise, and the term $| | A{y}_{t}-\tilde{x}| {| }_{2}^{2}$ captures the reconstruction error between the transformed latent variable *Ay*_*t*_ and the observation $\tilde{x}$. For our evaluation, we employed two common regularisers: *L*_1_, *L*_2_. These regularisers emphasise common characteristics of images, such as sparsity and smoothness.

We also investigate the influence of the parameter *η* on the learning process (Sup. Fig. 1). The parameter *η* acts as a weight for the learned gradient, influencing how it contributes to optimisation. We observe as the parameter increases that the metrics improve especially in the case of NRMSE. However, beyond *η* = 25, the performance metrics start worsening. This effect is because the learned gradient is not perfect, as the method implicitly has to learn the PSF *K*. Furthermore, since we are also using an approximation of ${\nabla }_{{y}_{t}}\log p(\tilde{x}| {y}_{t})$ which becomes worse as *t* approaches *T*, having a constant *η* will propagate errors further when obtaining the reconstruction.

## Discussion

Despite the immense progress in microscopy, the ability to visualise the microscopic world remains limited due to hardware imperfections and physics boundaries^8^. While recent advances in DL and generative models promise to assist in overcoming these barriers, these models come with their own set of limitations. DDPMs have shown great promise for generative modelling^30,51,52^. In applications such as biomedical image restoration and superresolution microscopy, the primary focus is on preventing hallucinations, eliminating artefacts, and ensuring the accuracy of the produced structures.

In this work, we demonstrate that incorporating the optical physical prior into the DDPM model enhances stability and yields more realistic reconstruction results. We achieve this by introducing a novel Physics-Informed Diffusion Model (PI-DDPM), a framework designed to enhance Diffusion Probabilistic Models (DDPM) by incorporating physics-based terms. We demonstrate meaningful improvements over the traditional DDPM framework by integrating domain-specific physical knowledge. Our detailed contributions are as follows. Firstly, we propose a method to incorporate a physics-informed (PI) term directly into the DDPM framework, enabling the model to account for domain-specific physical constraints during generation. This formulation is applicable to scenarios where physical knowledge, such as point spread functions (PSFs), is otherwise unavailable. Secondly, we demonstrate that our model can effectively learn the forward operator during training, leveraging this knowledge to apply a PI term dynamically during inference. This allows the model to remain physics-aware even when key imaging parameters are unknown, improving interpretability and robustness. Finally, we introduce a regularisation strategy for the inference process that consistently enhances performance compared to traditional DDPM approaches. Our results show that adding the PI term and regularising the inference process leads to improvements in model accuracy and consistency, especially in scenarios with limited data or high noise.

While the PI-DDPM we propose may not outperform specialist models like DFGAN^28^, it significantly outperforms its baseline DDPM in various metrics and in producing outputs closer to GT. Using various benchmarks and real datasets, we demonstrate that introducing a physical prior lowers the hallucinations of generative models like DDPM. Our approach extends a recently introduced paradigm of PI neural networks^36^ and extends the applicability of these methods to microscopy. Furthermore, since our model learns the distribution from the data, it estimates the mean and variance of the obtained reconstructions directly. This offers a convenient way to obtain confidence in the reconstructions, potentially facilitating broader adoption by the biomedical community.

## Methods

### Denoising diffusion probabilistic models

Unconditioned DDPMs start by setting a data distribution *y*_0_ ~ *q*(*y*_0_) and a Markovian noising process *q*, which incrementally injects noise, resulting in noised samples *y*_1_ to *y*_*T*_. Each step of the noising process introduces Gaussian noise according to a variance schedule denoted by *β*_*t*_:

18 $$q({y}_{t}| {y}_{t-1})=N({y}_{t};\sqrt{1-{\beta }_{t}}{y}_{t-1},{\beta }_{t}I)$$

It is unnecessary to apply *q* repeatedly to sample from *y*_*t*_ ~ *q*(*y*_*t*_∣*y*_0_). Instead, *q*(*y*_*t*_∣*y*_0_) can be represented as a Gaussian distribution. Defining *α*_*t*_ = 1 − *β*_*t*_ and ${\gamma }_{t}={\prod }_{s = 0}^{t}{\alpha }_{s}$, we have:

19 $$q({y}_{t}| {y}_{0})=N({y}_{t};\sqrt{{\gamma }_{t}}{y}_{0},(1-{\gamma }_{t})I)$$

20 $$=\sqrt{{\gamma }_{t}}{y}_{0}+\epsilon \sqrt{1-{\gamma }_{t}},\epsilon \sim N(0,I)$$

Here, 1 − *γ*_*t*_ indicates the noise variance at any given time step, which could also define the noise schedule instead of *β*_*t*_.

Applying Bayes’ theorem, the posterior *q*(*y*_*t*−1_∣*y*_*t*_, *y*_0_) is also a Gaussian with mean ${\tilde{\mu }}_{t}({y}_{t},{y}_{0})$ and variance ${\tilde{\beta }}_{t}$:

21 $${\tilde{\mu }}_{t}({y}_{t},{y}_{0})=\frac{\sqrt{{\gamma }_{t-1}}{\beta }_{t}}{1-{\gamma }_{t}}{y}_{0}+\frac{\sqrt{{\alpha }_{t}}(1-{\gamma }_{t-1})}{1-{\gamma }_{t}}{y}_{t}$$

22 $${\tilde{\beta }}_{t}=\frac{1-{\gamma }_{t-1}}{1-{\gamma }_{t}}{\beta }_{t}$$

23 $$q({y}_{t-1}| {y}_{t},{y}_{0})=N({y}_{t-1};\tilde{\mu }({y}_{t},{y}_{0}),{\tilde{\beta }}_{t}I)$$

To sample from the data distribution *q*(*y*_0_), one begins with *q*(*y*_*T*_) and sequentially samples reverse steps *q*(*y*_*t*−1_∣*y*_*t*_) until reaching *y*_0_. Given appropriate settings for *β*_*t*_ and *T*, *q*(*y*_*T*_) approximates an isotropic Gaussian, making the sampling of *y*_*T*_ straightforward. The subsequent step involves approximating *q*(*y*_*t*−1_∣*y*_*t*_) using a neural network to estimate its parameters: mean *μ*_*θ*_ and a covariance matrix *Σ*_*θ*_:

24 $${p}_{\theta }({y}_{t-1}| {y}_{t})=N({y}_{t-1};{\mu }_{\theta }({y}_{t},t),{\Sigma }_{\theta }({y}_{t},t))$$

To ensure *p*(*y*_0_) aligns with the true data distribution *q*(*y*_0_), one can optimise the variational lower-bound *L*_*vlb*_ for *p*_*θ*_(*y*_0_):

$${L}_{vlb}={L}_{0}+{L}_{1}+\ldots +{L}_{T-1}+{L}_{T}$$

$${L}_{0}=-\log {p}_{\theta }({y}_{0}| {y}_{1})$$

$${L}_{t-1}={D}_{{{{\rm{KL}}}}}(q({y}_{t-1}| {y}_{t},{y}_{0})| | {p}_{\theta }({y}_{t-1}| {y}_{t}))$$

$${L}_{T}={D}_{{{{\rm{KL}}}}}(q({y}_{T}| {y}_{0})| | p({y}_{T}))$$

However, an alternative objective yields better practical results. Instead of directly parameterizing *μ*_*θ*_(*y*_*t*_, *t*) as a neural network, they trained a model *ϵ*_*θ*_(*y*_*t*_, *t*) to predict *ϵ* from the aforementioned equation. The simplified objective is defined as:

25 $${L}_{{{{\rm{simple}}}}}={E}_{t \sim [1,T],{y}_{0} \sim q({y}_{0}),\epsilon \sim N(0,I)}[| | \epsilon -{\epsilon }_{\theta }({y}_{t},t)| {| }^{2}]$$

For sampling, *μ*_*θ*_(*y*_*t*_, *t*) can be derived from *ϵ*_*θ*_(*y*_*t*_, *t*) via substitution:

26 $${\mu }_{\theta }({y}_{t},t)=\frac{1}{\sqrt{{\alpha }_{t}}}\left({y}_{t}-\frac{{\beta }_{t}}{\sqrt{1-{\gamma }_{t}}}{\epsilon }_{\theta }({y}_{t},t)\right)$$

and sample using the following recurrence:

$${y}_{t-1}={\mu }_{\theta }({y}_{t},t)+\sqrt{{\Sigma }_{\theta }}{\epsilon }_{t},\quad {\epsilon }_{t} \sim {{{\mathcal{N}}}}(0,I)$$

It is feasible to fix *Σ*_*θ*_(*y*_*t*_, *t*) to a constant value, such as *β*_*t*_*I* or ${\tilde{\beta }}_{t}I$, representing upper and lower bounds for the true reverse step variance, instead of learning the covariance^30^.

### Conditioned denoising probabilistic diffusion models

In the conditioned case, samples are drawn from an unknown distribution *p*(*y*∣*x*). This is referred to as distribution because conditioned image synthesis is, by nature, ill-posed. Specifically, there are many possible solutions *y* for any given input *x*. In the conditioned process, we want to learn an approximation of this distribution. To achieve that, it is possible to condition DDPMs in two ways.

The first way is to redefine the Markov chain. Given an image *y* and some corruption process *p*(*x*∣*y*), we want to learn *p*(*y*∣*x*). To achieve this, the diffusion Markov chain states are concatenated by the respective conditioning image *x*^32^. Specifically, the distributions of the states of the Markov chain are generated by the diffusion process *q*(*y*_*t*_∣*y*_*t*−1_) and concatenated to the conditioning image *x*. Where ${y}_{i}=\sqrt{{\gamma }_{t}}{y}_{0}+\epsilon \sqrt{1-{\gamma }_{t}},\epsilon \sim {{{\mathcal{N}}}}(\epsilon ;0,I)$. To learn the reverse process, a reverse Markov chain is established, where $p({y}_{T})={{{\mathcal{N}}}}({y}_{T};0,I)$:

27 $${p}_{\theta }({y}_{t-1}| {y}_{t},x)={{{\mathcal{N}}}}({y}_{t-1}| {\mu }_{\theta }(x,{y}_{t},{\gamma }_{t}),{\sigma }_{t}^{2}I).$$

Using the same formulation as in regular DDPMs^32^, the model is trained to predict *ϵ* at each time step:

28 $$\mathop{\min }_{\theta }{L}_{{{{\rm{simple}}}}}:= {{\mathbb{E}}}_{t,(x,{y}_{0}),\epsilon }| | \epsilon -{\epsilon }_{\theta }({y}_{t},x,{\gamma }_{t})| {| }_{2}^{2}.$$

Finally, to obtain *y*_0_, the same iterative denoising as in the regular DDPMs is applied.

29 $${y}_{t-1}=\frac{1}{\sqrt{{\alpha }_{t}}}\left({y}_{t}-\frac{{\beta }_{t}}{\sqrt{1-{\gamma }_{t}}}{\epsilon }_{\theta }(x,{y}_{t},{\gamma }_{t})\right)+\sqrt{{\beta }_{t}}{\epsilon }_{t}.$$

### Model-guided denoising probabilistic diffusion models

The second way to obtain conditioned samples from a diffusion model is to condition an unconditioned reverse process^33^. Given an unconditional reverse process *p*_*θ*_(*y*_*t*_∣*y*_*t*+1_), to condition on label *x* we can factorise

30 $${p}_{\theta ,\phi }({y}_{t}| {y}_{t+1},x)=Z{p}_{\theta }({y}_{t}| {y}_{t+1}){p}_{\phi }(x| {y}_{t}),$$

where *Z* is a normalizing constant. This expression can be approximated as a perturbed Gaussian distribution. Since our unconditioned reverse process is a Gaussian, we have:

31 $${p}_{\theta }({y}_{t}| {y}_{t+1})={{{\mathcal{N}}}}(\mu ,{\sigma }_{t}^{2}I)$$

32 $$\log {p}_{\theta }({y}_{t}| {y}_{t+1})=-\frac{1}{2{\sigma }_{t}^{2}}{({y}_{t}-\mu )}^{T}({y}_{t}-\mu )+C.$$

Since, at infinity, the distribution of the reverse process tends to a delta distribution, then it is reasonable to approximate *p*_*ϕ*_(*x*∣*y*_*t*_) by its Taylor expansion around the mean.

33 $$\log {p}_{\phi }(x| {y}_{t})\approx \log {p}_{\phi }(x| {y}_{t}){| }_{{y}_{t} = \mu }+({y}_{t}-\mu ){\nabla }_{{y}_{t}} \\ \log {p}_{\phi }(x| {y}_{t}){| }_{{y}_{t} = \mu } =({y}_{t}-\mu )g+{C}_{1}$$

where $$g={\nabla }_{{y}_{t}}\log {p}_{\phi }(x| {y}_{t})$$

Finally, by replacing and rearranging, we get:

34 $${p}_{\theta }({y}_{t}| {y}_{t+1}){p}_{\phi }(x| {y}_{t})\approx {{{\mathcal{N}}}}(\mu +{\sigma }_{t}^{2}g,{\sigma }_{t}^{2}I).$$

Thus, the reverse conditioned process approximates the unconditioned Gaussian transition with its mean shifted by ${\sigma }_{t}^{2}g$.

### Physics-informed denoising probabilistic diffusion models training

We trained our PI-DDPM employing the algorithm DDPM as outlined in Algorithm 1. For inference, we used the algorithm outline in Algorithm 2.

#### Algorithm 1

Physics-Informed Denoising Diffusion Model (PI-DDPM) Training



#### Algorithm 2

Physics-Informed Denoising Diffusion Model (PI-DDPM) Sampling



### Metrics

To assess the performance of all the models in this study we used multi-scale structural similarity index measure (MS-SSIM)^46^, normalised root mean square error $\,{\mbox{NRMSE}}:= \frac{\sqrt{{\mbox{MSE}}}}{{y}_{{{{\rm{max}}}}}-{y}_{{{{\rm{min}}}}}}$, and peak single-to-noise ratio (PSNR). MS-SSIM metric is defined as:

35 $$\,{{\mbox{MS-SSIM}}}\,(x,y):= {[{l}_{M}(x,y)]}^{{\alpha }_{M}}{\prod }_{j=1}^{M}{[{c}_{j}(x,y)]}^{{\beta }_{j}}{[{s}_{j}(x,y)]}^{{\gamma }_{j}},$$

where *l*_*j*_, *c*_*j*_, and *s*_*j*_ are the measures of luminance, contrast, and structure corresponding to scale j. We used five scales and *α*_*j*_ = *β*_*j*_ = *γ*_*j*_ for $\mathop{\sum }_{j = 1}^{M}{\gamma }_{j}=1$ in accordance with the parameters reported in^46^. PSNR was defined as: $\,{{\mbox{PSNR}}}:= 20{\log }_{10}({{\mbox{MAX}}}_{I})-10{\log }_{10}({\mbox{MSE}}),$ where MAX_*I*_ is the maximum pixel value of the image and MSE is the mean square error.

### Training details

DDPM and PI-DDPM were trained in a cluster environment employing a single Nvidia A100 with 40GB of vRAM. The batch size was 16. Pre-training continued for 1 million iterations using an ImageNet-derived dataset and fine-tuned for 800,000 iterations on the mixed dataset. The U-Net model was trained in a cluster environment using a single Nvidia V100 GPU equipped with 32GB of vRAM. Pre-training continued for 800,000 iterations, and fine-tuning for 100,000.

### Simulated dataset generation

To generate a simulated dataset for training our models, we utilised two primary sources of images: photographs from the ImageNet database^44^ and structured illumination microscopy (SIM) images from the BioSR dataset^29^. Our approach involved processing each image through a forward microscopy model, termed the Image Acquisition Model (see Methods and Fig. 1c). This model simulates the effects of different microscopes by convolving each image with a Point Spread Function (PSF) and then applying Poisson noise.

For the ImageNet-derived dataset, which comprises 1.2 million training images and 100,000 test images, we generated 30,000 unique PSFs. The generation of each PSF involved random sampling of physically plausible parameters for microscopy systems: numerical aperture (0.4 to 1.0), excitation wavelength (320 to 400 *μ*m), emission wavelength (450 to 550 *μ*m), and pinhole size (0.1 to 1000 *μ*m). The focal plane was centered in the volume object with a refractive index of 1.33, corresponding to air. We then convolved the ImageNet images with these PSFs to create a training dataset that mimics microscopy images.

Additionally, we incorporated a simulated dataset derived from the publicly available BioSR dataset^29^. BioSR contains approximately 20,000 SIM fluorescence images of various subcellular structures, such as clathrin-coated pits (CCPs), endoplasmic reticulum (ER), microtubules (MT), and F-actin, imaged at different fluorescence levels. For this part of the dataset, we took all high-resolution images from BioSR and processed them using the Image Acquisition Model to simulate low-resolution images. These simulated BioSR images, adhering to the original dataset’s train-test split, were used for fine-tuning our models. The high-resolution BioSR images served as the ground truth during training, while the simulated low-resolution images were used as input. At test time, we employed BioSR’s low-resolution (widefield) images as inputs.

This combined, processed dataset from ImageNet and BioSR, which includes both training and test images, was employed for training and fine-tuning various models, including U-Net^27^, DDPM^30^, and our proposed PI-DDPM model. The goal was to reconstruct high-resolution images from the simulated blurred micrographs.

### Direct stochastic optical reconstruction microscopy dataset

To test how our method compares to the state-of-the-art single-molecule localisation microscopy (SMLM) we have employed a publicly available three-color Direct Stochastic Optical Reconstruction Microscopy (dSTORM) dataset^49^. In this dataset authors provide a Widefield and SMLM reconstructed high-resolution image containing mid-zygotene nucleus immunostained for SYCP3 (red), DMC1 (green) and RAD51 (blue) proteins. Images in this dataset were acquired by Zeiss Elyra PS1 microscope using a 100x 1.46NA oil immersion objective. Further imaging details are provided by the authors in the following publication^53^.

### Prospective correlative widefield-confocal microscopy dataset

Finally, to test how our model would perform on a prospectively acquired dataset, we have obtained a correlated widefield-confocal microscopy dataset. For this, A549 lung carcinoma cell line cells were seeded in 96-well imaging plates a night prior to imaging, then fixed with 4% paraformaldehyde (Sigma) and stained for DNA with Hoechst 33342 fluorescent dye (Sigma). Cell culture was maintained similarly to the procedures described in^54^. Next, stained cell nuclei were imaged using ImageXpress Confocal system (Molecular Devices) in either confocal or widefield mode employing Nikon 20X Plan Apo Lambda objective. To obtain 3D information images in both modes were acquired as Z-stacks with 0.3 *μ*m and 0.7 *μ*m for confocal and widefield modes respectively. Confocal z-stack was Nyquist sampled. The excitation wavelength was 405 nm and the emission 452 nm. Using these settings we have obtained 72 individual stacks for both modalities, with each stack covering 2048 by 2048 pixels or 699 by 699 *μ*m.

### Widefield to SIM (W2S) dataset

Widefield to SIM (W2S)^47^ is a collection obtained through conventional fluorescence widefield and SIM (Structured Illumination Microscopy) imaging techniques. This dataset encompasses 144,000 authentic fluorescence microscopy images, aggregating into a total of 360 distinct image sets. Each set consists of low-resolution (LR) widefield images exhibiting various levels of noise, an accompanying noise-free LR image, and a corresponding high-resolution (HR) SIM image of superior quality.

### Source code

The source code for this work is available on GitHub (see Code availability). To train the model place your data generated by the dataset_generation script (if you are generating simulated data) or the STORM script if you are generating the respective dSTORM dataset. In the train_ddpm or train_unet script change the paths of the loading data to the ones that you generated. Next, choose a training modality, either widefield or confocal. Finally, run the script.

To test the model generate your testing dataset using the dataset_generation script. Change the paths corresponding to your data. Next, change the paths to the weights files that you wish to use. Finally, run the test script. Due to the size limitation of the submission for the prospective dataset containing correlative widefield-confocal fluorescence microscopy, we provide only a single stack as an example (data/teaser_c_w_test.npz).

## Supplementary information

Supplementary Material

nr-reporting-summary

## Data Availability

The correlated widefield confocal microscopy dataset acquired prospectively in this work is available under a Creative Commons license on the RODARE data repository^50^. All other datasets used in this work are published and available from their respective sources.

## Code availability

The source code for this work is available on GitHub under GNU General Public License v3.0 and can be accessed at^55^ and https://github.com/casus/pi-ddpm. Additionally, the code for decorrelation-based resolution measurement can be found in^48^ and https://github.com/casus/pyres. See Methods for details required to reproduce the results.
