# Supplementary material for: Microscopy image reconstruction with physics-informed denoising diffusion probabilistic model
Source: Commun Eng. 2024 Dec 29;3:186. doi: 10.1038/s44172-024-00331-z (PMC11683148; doi:10.1038/s44172-024-00331-z)
Supplement: Supplementary file 1 — Supplementary Material [file 44172_2024_331_MOESM1_ESM.pdf]

Supplementary information to the manuscript: Microscopy image reconstruction with physics-informed denoising diffusion probabilistic model

Authors: Rui Li*, Gabriel della Maggiora*, Vardan Andriasyan, Anthony Petkidis, Artsemi Yushkevich, Nikita Deshpande, Mikhail Kudryashev, Artur Yakimovich
*these authors contributed equally to this work

correspondence: a.yakimovich@hzdr.de

Supplementary Results

Performance on BioSR Microscopy Dataset

Table S1. Performance on BioSR Structures. **Best**, <u>second best</u>.

(a) CCP structures.

Metric / Model	DFCAN	DFGAN	DDPM	PI-DDPM (ours)
MS-SSIM (↑)	0.945	**0.975**	0.825	<u>0.943</u>
MAE (↓)	0.005	**0.004**	0.040	<u>0.005</u>
Resolution (nm) (↓)	107	**97**	102	<u>98</u>

(b) ER structures.

Metric / Model	DFCAN	DFGAN	DDPM	PI-DDPM (ours)
MS-SSIM (↑)	0.820	**0.963**	0.926	<u>0.960</u>
MAE (↓)	0.047	**0.029**	0.039	<u>0.035</u>
Resolution (nm) (↓)	163	**144**	159	<u>155</u>

(c) MT structures.

Metric / Model	DFCAN	DFGAN	DDPM	PI-DDPM (ours)
MS-SSIM (↑)	0.813	**0.894**	0.862	<u>0.881</u>
MAE (↓)	0.054	**0.033**	0.041	<u>0.035</u>
Resolution (nm) (↓)	121	**113**	120	<u>115</u>

(d) F-actin structures.

Metric / Model	DFCAN	DFGAN	DDPM	PI-DDPM (ours)
MS-SSIM (↑)	**0.906**	<u>0.904</u>	0.870	0.884
MAE (↓)	<u>0.032</u>	**0.031**	0.041	0.036
Resolution (nm) (↓)	130	<u>112</u>	113	**105**

Performance on BioSR Microscopy Dataset under Varying Noise Conditions

To assess the robustness of our proposed PI-enhanced DDPM model under conditions typical of challenging microscopy scenarios, we conducted an experiment focused on limited data availability and high noise levels. Using the F-actin class images from the bioSR dataset, we randomly sampled 100 images from the test set and simulated low-quality widefield acquisitions by introducing controlled noise. Specifically, we applied an approximate Poisson-Gaussian noise model with variance levels in the range $\xi = 0.025$ to $\xi = 0.05$, where ξ corresponds to the Gaussian noise component $N(\xi, \xi)$. These settings replicate real-world imaging conditions often seen in microscopy, where signal degradation occurs due to environmental factors or acquisition constraints.

Reconstruction fidelity was evaluated through Multi-Scale Structural Similarity Index Measure (MS-SSIM), Mean Absolute Error (MAE), and resolution comparisons against ground truth. For each metric, we averaged the errors across the 100 randomly sampled images and plotted the results to provide a comprehensive view of model performance, as shown in Figure S2. The PI-DDPM model consistently achieved higher MS-SSIM scores, lower MAE, and improved resolution, outperforming the standard DDPM model across all noise levels. Qualitative analysis (Figure S2) further demonstrates that our model retained fine structural details in high-noise conditions where standard DDPM reconstructions showed degradation. These findings underscore the PI-DDPM model's effectiveness in maintaining accuracy under data-scarce and high-noise environments, confirming its utility in real-world applications such as low-signal microscopy where traditional models often underperform.

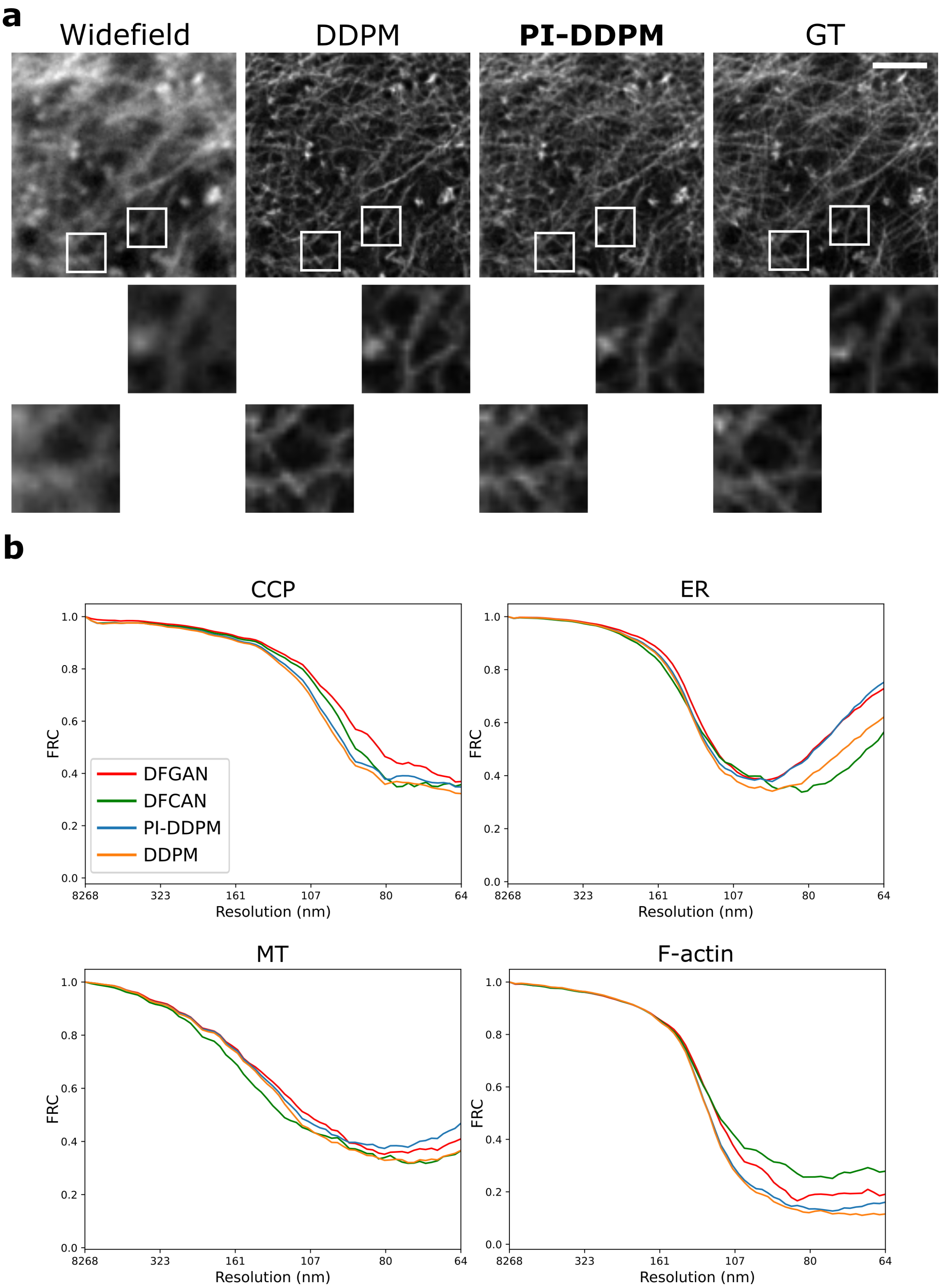

Figure S1. **Comparison of model performance BioSR-derrived dataset**. **a**, Denoising Diffusion Probabilistic Models (DDPM) and physics-informed DDPM (PI-DDPM) in BioSR images. GT stands for ground truth. MT stands for microtubules. The scale bar in micrographs is 2 μm. **b**, Fourier Ring Correlation (FRC) for different structures in the BioSR dataset: clathrin-coated pits (CCP), endoplasmic reticulum (ER), microtubules (MT) and F-actin.

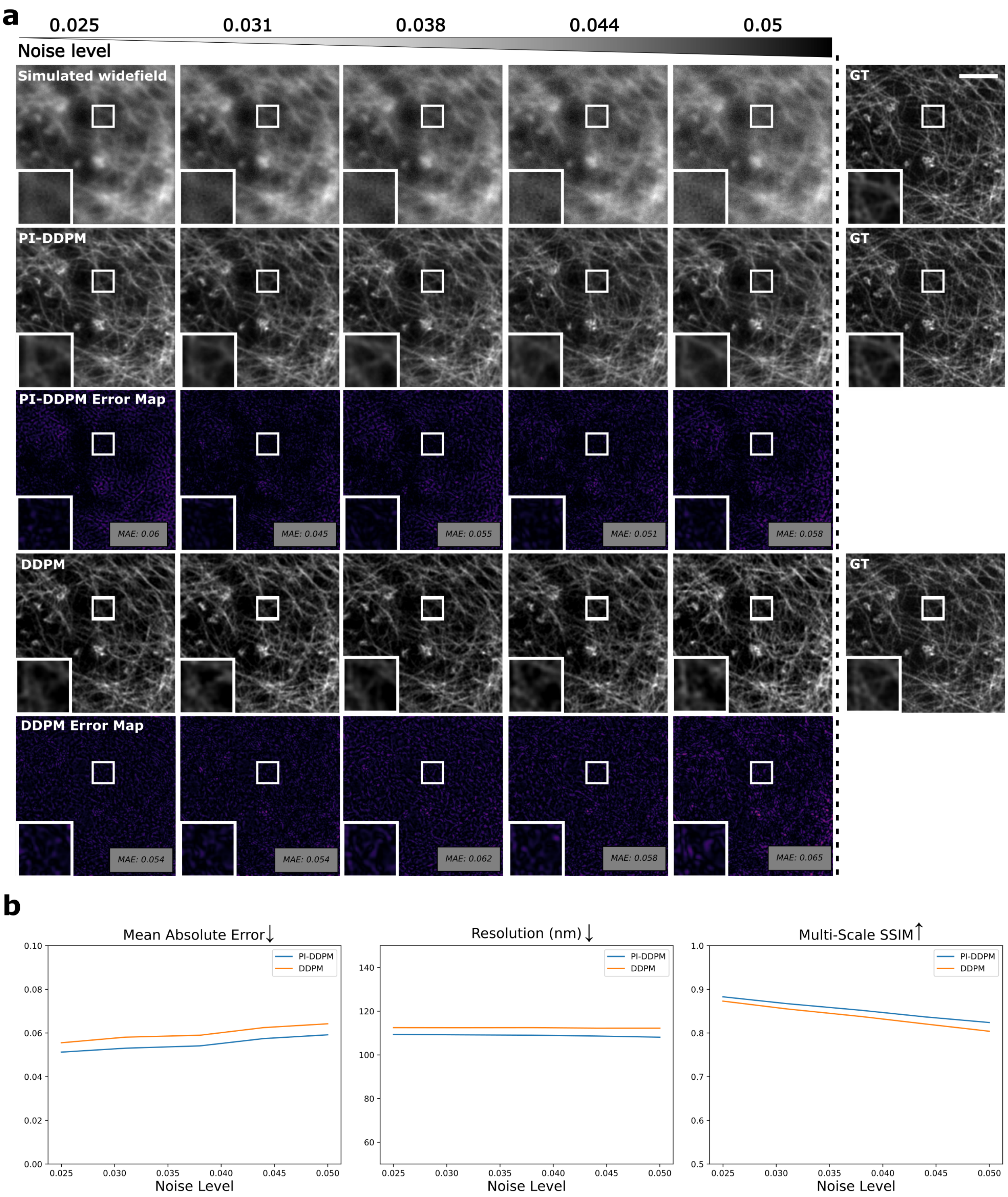

Figure S2. **Comparison of model performance on BioSR-derived dataset under varying amount of synthetic noise**. **a**, Denoising Diffusion Probabilistic Models (DDPM) and physics-informed DDPM (PI-DDPM) on limited samples of F-actin from BioSR images. GT stands for ground truth. The scale bar is 2 μm. **b**, Comparison of averaged metrics over different noise levels.

Ablation study

Method	Regulariser Weight (L_1)				
	0	0.001	0.01	0.1	1
PI-DDPM ($\eta = 10$)	0.886	0.885	0.887	0.887	0.83
DDPM	0.866	0.866	0.875	0.879	0.83

(a) Ablations Regularisers MS-SSIM Metric

Method	Regulariser Weight (L_1)				
	0	0.001	0.01	0.1	1
PI-DDPM ($\eta = 10$)	0.05	0.051	0.051	0.054	0.109
DDPM	0.061	0.062	0.057	0.072	0.11

(b) Ablations for Regularisers NRMSE Metric

Table S2. Ablation Studies for L_1 Regularisers with Different Metrics

Method	Regulariser Weight (L_2)				
	0	0.001	0.01	0.1	1
PI-DDPM ($\eta = 10$)	0.885	0.887	0.891	0.891	0.864
DDPM	0.866	0.866	0.874	0.88	0.862

(a) Ablations for Regularisers MS-SSIM Metric

Method	Regulariser Weight (L_2)				
	0	0.001	0.01	0.1	1
PI-DDPM ($\eta = 10$)	0.052	0.051	0.049	0.055	0.085
DDPM	0.063	0.063	0.059	0.065	0.091

(b) Ablations for Regularisers NRMSE Metric

Table S3. Ablation Studies for L_2 Regularisers with Different Metrics
