# Supplementary material for: Microscopy image reconstruction with physics-informed denoising diffusion probabilistic model
Source: Commun Eng. 2024 Dec 29;3:186. doi: 10.1038/s44172-024-00331-z (PMC11683148; doi:10.1038/s44172-024-00331-z)
Supplement: Supplementary file 2 — nr-reporting-summary [file 44172_2024_331_MOESM2_ESM.pdf]

nature portfolio

Corresponding author(s): Artur Yakimovich

Last updated by author(s): Nov 19, 2024

Reporting Summary

Nature Portfolio wishes to improve the reproducibility of the work that we publish. This form provides structure for consistency and transparency in reporting. For further information on Nature Portfolio policies, see our Editorial Policies and the Editorial Policy Checklist.

Statistics

For all statistical analyses, confirm that the following items are present in the figure legend, table legend, main text, or Methods section.

n/a	Confirmed	
☐	☒	The exact sample size (*n*) for each experimental group/condition, given as a discrete number and unit of measurement
☐	☒	A statement on whether measurements were taken from distinct samples or whether the same sample was measured repeatedly
☒	☐	The statistical test(s) used AND whether they are one- or two-sided<br>*Only common tests should be described solely by name; describe more complex techniques in the Methods section.*
☒	☐	A description of all covariates tested
☒	☐	A description of any assumptions or corrections, such as tests of normality and adjustment for multiple comparisons
☒	☐	A full description of the statistical parameters including central tendency (e.g. means) or other basic estimates (e.g. regression coefficient) AND variation (e.g. standard deviation) or associated estimates of uncertainty (e.g. confidence intervals)
☒	☐	For null hypothesis testing, the test statistic (e.g. *F*, *t*, *r*) with confidence intervals, effect sizes, degrees of freedom and *P* value noted<br>*Give P values as exact values whenever suitable.*
☒	☐	For Bayesian analysis, information on the choice of priors and Markov chain Monte Carlo settings
☒	☐	For hierarchical and complex designs, identification of the appropriate level for tests and full reporting of outcomes
☒	☐	Estimates of effect sizes (e.g. Cohen's *d*, Pearson's *r*), indicating how they were calculated

Our web collection on statistics for biologists contains articles on many of the points above.

Software and code

Policy information about availability of computer code

Data collection	The correlated widefield confocal microscopy dataset acquired prospectively in this work is available under a Creative Commons license on the RODARE data repository https://doi.org/10.14278/rodare.2668. All other datasets used in this work are published and available from their respective sources (cited in the manuscript).
Data analysis	The source code for this work is available on GitHub under GNU General Public License v3.0 and can be accessed at [1] and https://github.com/casus/pi-ddpm. Additionally, the code for decorrelation-based resolution measurement can be found in [2] and https://github.com/casus/pyres. See Methods for details required to reproduce the results. (1) Li, R. et al. casus/pi-ddpm: Initial release, DOI: https://doi.org/10.5281/zenodo.10406506 (2023).467 2) Deshpande, N. & Yakimovich, A. Pyres: python-based decorrelation analysis for resolution estimation, DOI: https://doi.org/10.5281/zenodo.12699495 (2024).455]

For manuscripts utilizing custom algorithms or software that are central to the research but not yet described in published literature, software must be made available to editors and reviewers. We strongly encourage code deposition in a community repository (e.g. GitHub). See the Nature Portfolio guidelines for submitting code & software for further information.

Data

Policy information about availability of data

All manuscripts must include a data availability statement. This statement should provide the following information, where applicable:

- Accession codes, unique identifiers, or web links for publicly available datasets
- A description of any restrictions on data availability
- For clinical datasets or third party data, please ensure that the statement adheres to our policy

The correlated widefield confocal microscopy dataset acquired prospectively in this work is available under a Creative Commons license on the RODARE data repository https://doi.org/10.14278/rodare.2668. All other datasets used in this work are published and available from their respective sources.

Human research participants

Policy information about studies involving human research participants and Sex and Gender in Research.

Reporting on sex and gender	n/a
Population characteristics	n/a
Recruitment	n/a
Ethics oversight	n/a

Note that full information on the approval of the study protocol must also be provided in the manuscript.

Field-specific reporting

Please select the one below that is the best fit for your research. If you are not sure, read the appropriate sections before making your selection.

☒ Life sciences ☐ Behavioural & social sciences ☐ Ecological, evolutionary & environmental sciences

For a reference copy of the document with all sections, see nature.com/documents/nr-reporting-summary-flat.pdf

Life sciences study design

All studies must disclose on these points even when the disclosure is negative.

Sample size	to facilitate an adequate dataset size all available date was used to train the models. The purpose of the study was not to elucidate biological mechanisms. Therefore, statistical power was not considered.
Data exclusions	datasets were used as is, without specific data exclusion strategy.
Replication	as the purpose of the study was not to elucidate biological mechanisms, only technical measurement replica were used.
Randomization	Randomization was an integral part of the deep learning training algorithms.
Blinding	Blinding was not applicable to this study.

Reporting for specific materials, systems and methods

We require information from authors about some types of materials, experimental systems and methods used in many studies. Here, indicate whether each material, system or method listed is relevant to your study. If you are not sure if a list item applies to your research, read the appropriate section before selecting a response.

Materials & experimental systems

n/a	Involved in the study
☒	☐ Antibodies
☐	☒ Eukaryotic cell lines
☒	☐ Palaeontology and archaeology
☒	☐ Animals and other organisms
☒	☐ Clinical data
☒	☐ Dual use research of concern

Methods

n/a	Involved in the study
☒	☐ ChIP-seq
☒	☐ Flow cytometry
☒	☐ MRI-based neuroimaging

Eukaryotic cell lines

Policy information about cell lines and Sex and Gender in Research

Cell line source(s)	A549 (ATCC)
Authentication	cell lines were kept according to bona fide cell bank standards upon arrival from the ATCC source. No further authentication was performed.
Mycoplasma contamination	cells were kept at a low passage and regularly checked for mykoplasma contamination.
Commonly misidentified lines (See ICLAC register)	the cell line is not a part of the ICLAC v13 (current to date)
